# The Role of cccDNA in HBV Maintenance

**DOI:** 10.3390/v9060156

**Published:** 2017-06-21

**Authors:** Lena Allweiss, Maura Dandri

**Affiliations:** 1 I. Department of Internal Medicine, Center for Internal Medicine, University Medical Center Hamburg-Eppendorf, Martinistr. 52, D-20246 Hamburg, Germany; l.allweiss@uke.de; 2 German Center for Infection Research (DZIF), Hamburg-Lübeck-Borstel Site, Germany

**Keywords:** hepatitis B virus, cccDNA, animal models, human liver chimeric mice, cell proliferation

## Abstract

Chronic hepatitis B virus (HBV) infection continues to be a major health burden worldwide; it can cause various degrees of liver damage and is strongly associated with the development of liver cirrhosis and hepatocellular carcinoma. The molecular mechanisms determining HBV persistence are not fully understood, but these appear to be multifactorial and the unique replication strategy employed by HBV enables its maintenance in infected hepatocytes. Both the stability of the HBV genome, which forms a stable minichromosome, the covalently closed circular DNA (cccDNA) in the hepatocyte nucleus, and the inability of the immune system to resolve chronic HBV infection are believed to be key mechanisms of HBV chronicity. Since a true cure of HBV requires clearance of intranuclear cccDNA from infected hepatocytes, understanding the mechanisms involved in cccDNA biogenesis, regulation and stability is mandatory to achieve HBV eradication. This review will summarize the state of knowledge on these mechanisms including the impact of current treatments on the cccDNA stability and activity. We will focus on events challenging cccDNA persistence in dividing hepatocytes.

## 1. Introduction

Liver disease associated to persistent infection with hepatitis B virus (HBV) continues to be a major health problem of global impact. Even if HBV is not directly cytopathic for the infected cell, the infection leads to a wide spectrum of liver disease spanning from acute resolving to chronic infection with different grades of hepatitis, which often progresses to liver cirrhosis and hepatocellular carcinoma [1]. In spite of the existence of an effective vaccine, at least 240 million people are estimated to be chronically infected worldwide and treatments enabling to cure chronic HBV infection are currently not available. Chronic infection with HBV is characterized by the persistence of the episomal viral genome, the covalently closed circular DNA (cccDNA), which forms a stable minichromosome in the nuclei of infected hepatocytes. Both the longevity of the cccDNA and the inability of the immune system to mount effective immune responses against the virus appear responsible for the failure of viral clearance and relapse after treatment cessation. Thus, to achieve a true cure of chronic HBV infection, cccDNA needs to be eliminated from infected hepatocytes. Understanding the role of the cccDNA in HBV maintenance, learning about cccDNA formation, its transcriptional regulation, turnover and intracellular stability will be fundamental to find a cure.

## 2. HBV Infection and cccDNA Establishment

The hepatitis B virus is a small blood-born pathogen transmitted by percutaneous exposure to infected blood or body fluids. Through the bloodstream, the virus reaches the liver to infect the hepatocytes, which are the only target cells susceptible for infection. Thus, characteristic of HBV is its high tissue and species specificity, as well as a unique genomic organization and replication mechanism. Upon cell entry, which involves an irreversible binding of the virion to the hepatocyte-specific receptor, the Na^+^-taurocholate cotransporting polypeptide (NTCP) [2], the viral genome needs to be transferred to the hepatocyte nuclei to establish a productive infection. Of note, the steps following viral entry are still poorly characterized. However, in vitro studies showed that they involve endocytosis and microtubule-mediated transport of the nucleocapsids to the nuclear envelope [3]. Through interactions with nuclear transport receptors and adaptor proteins of the nuclear pore complex, the capsids eventually disintegrate permitting the release of both core capsid subunits and of the relaxed circular HBV DNA (rcDNA) genome [4].

Although the mechanisms permitting the conversion of the incoming rcDNA form to the supercoiled cccDNA molecule remain largely unknown, they appear to take place via a multi-step process and to require the cellular DNA repair machinery [5,6]. First, the covalently attached viral polymerase needs to be removed, a process leading to the formation of a protein-free rcDNA (PF-rcDNA) intermediate that may even persist in infected cells [7]. CccDNA synthesis further requires the removal of an RNA primer from the positive strand, removal of terminally redundant sequences from the negative strand, and repair of the incomplete positive strand before both DNA strands are ligated [8]. Thus, the establishment of the episomal, plasmid-like form of the HBV genome relies on specific interactions with distinct cellular components, such as the DNA polymerase κ which was shown to contribute substantially to the completion of the positive strand DNA in rcDNA [9]. Another study demonstrated that the cellular DNA repair enzyme tyrosyl-DNA-phosphodiesterase 2 (TDP2) can release the covalently bound viral polymerase from HBV and duck hepatitis B virus (DHBV) rcDNA in vitro, and a physiological role in cccDNA formation was suggested because human cells with a stable knock down of TDP2 expression significantly slowed down DHBV rcDNA to cccDNA conversion. This phenotype was confirmed in *TDP2* gene knockout cells [10]. However, human HBV was still able to infect a susceptible version of these knockout cells, indicating that TDP2 is at least not essential for HBV cccDNA formation [11]. Identifying all host factors determining the dynamics of cccDNA formation as well as HBV entry will be thus crucial to progress in the development of curative therapeutic approaches.

## 3. CccDNA Activity and Pool Size

Within the nucleus, the cccDNA interacts with histone and non-histone proteins resembling cellular chromatin [12]. In the same way as gene transcription is regulated in host chromatin, cccDNA transcription, which is under control of two enhancer elements and four distinct promoters, relies on the activity and dynamic interplay of numerous transcription factors, co-activators, co-repressors and chromatin modifying enzymes [13]. The cccDNA bears numerous binding sites for ubiquitous and liver-specific transcription factors and transfection studies in hepatoma cell lines indicated their involvement in the transcription of viral RNAs [14]. Nevertheless, knowledge of the molecular mechanisms regulating cccDNA activity in infected primary hepatocytes is still limited. In hepatoma cell lines, cccDNA transcription seems to be regulated by the acetylation status of cccDNA-bound H3 and H4 histones and, indeed, in HBV-infected patients, histone hypoacetylation and histone deacetylase 1 recruitment onto the cccDNA correlates with low HBV viremia [15]. Using a sophisticated chromatin immunoprecipitation sequencing approach, Tropberger and colleagues mapped post-translational histone modifications across the entire HBV genome in HBV-infected HepG2-NTCP cells, primary human hepatocytes and liver biopsies revealing an unusual chromatin organization [16]. Even though distribution and levels of active histone modifications are comparable to cellular chromatin and are enriched at HBV promotors, there is an underrepresentation of repressive marks even at silent promotors. Given the remarkably different organization of the viral genome—such as its circular conformation, its small size and compact organization of transcripts and regulatory elements—it is not surprising to find distinct differences also in its epigenetic regulation. The precise nature of these differences and whether its epigenetic regulation will be amenable to therapeutic intervention remains an open question. Apart from cellular factors, viral proteins were also shown to be associated with the cccDNA. The viral core protein appears to be a structural component of the cccDNA minichromosome responsible for the reduced nucleosomal spacing on the cccDNA compared to cellular chromatin [17]. Thus, core proteins might also be involved in the regulation viral transcription. In addition, the non-structural regulatory hepatitis B virus X protein (HBx) was shown to be recruited to the cccDNA minichromosome [18] and to be required to initiate cccDNA-driven transcription of the viral RNAs [19]. Moreover, recent studies showed that HBx mediates the degradation of the ‘structural maintenance of chromosomes’ (Smc) complex Smc5/6 [20,21]. By binding to the damaged DNA binding protein 1 (DDB1), HBx can promote the interaction of smc5/6 with a component of the ubiquitin proteasome system, the E3 ubiquitin ligase named Cul4, to trigger ubiquitination and degradation of the smc5/6 complex. Being involved in chromosome organization and DNA repair, the Smc5/6 complex, suspected of binding to the cccDNA, may act as a host restriction factor suppressing cccDNA transcription. Altogether, these studies show that not only the cellular transcriptional machinery but also the non-structural HBx protein plays a key role in cccDNA regulation.

Through the establishment of a cccDNA minichromosome in infected hepatocytes, viral replication may initially occur without raising the attention of intrinsic antiviral defense mechanisms [22,23,24]. The cccDNA is the template of transcription for five viral RNAs necessary for the production of the viral antigens and for viral replication, the latter of which takes place in the cytoplasm after reverse transcription of an over-length pregenomic RNA (pgRNA) within newly formed nucleocapsids [25]. Mature, rcDNA-containing nucleocapsids are then enveloped and secreted into the bloodstream as progeny viruses. HBV appears to have developed sophisticated strategies enabling the virus to camouflage its genome as a minichromosome, hijacking the cellular transcriptional machinery for its replication needs, and concealing the production of new virions inside the nucleocapsids and hence does not offer many possibilities to the host to recognize the infection.

The transcriptional activity of the cccDNA can be determined by measuring pgRNA contents in the liver. To avoid the need for liver biopsies, circulating antigens can serve as serum markers for cccDNA activity to assess disease progression or treatment response. Among them, core related antigen and viral RNA have become attractive candidates in the last years (as reviewed in [26]). In addition to DNA containing viral capsids, HBV RNA is present in the serum of chronically HBV-infected patients most likely as pregenomic RNA encapsidated and enveloped in virus-like particles. Serum pgRNA was shown to reflect the amount of pgRNA present in the whole liver and hence its amount in serum may serve as surrogate marker to estimate the presence of transcriptionally active covalently closed circular DNA [27,28,29].

Infection studies performed in ducks and woodchucks with their respective HBV-related hepadnaviruses (DHBV and woodchuck hepatitis virus—WHV), revealed that cccDNA may be also established from newly synthesized rcDNA-containing nucleocapsids that are imported into the cell nucleus to build up a cccDNA pool (intracellular recycling). It is worth noting that this mechanism of intracellular cccDNA amplification was shown to play a major role in the early phases of infection of duck and woodchuck hepatocytes, where a high copy number of cccDNA molecules is generally detected (1–17 molecules/cell in ducks [30] and up to 50 molecules/cell in woodchucks [31]). In contrast, in vitro studies indicated that in comparison to HBV-related viruses, cccDNA formation and intracellular cccDNA amplification are less efficient in human cells [32,33]. A sophisticated experimental study involving cross-species transfection experiments provided evidence that HBV converts the rcDNA into cccDNA less efficiently than DHBV in the same human cell background [33], suggesting that not only the host but also the virus itself controls cccDNA dynamics and cccDNA pool size in infected human hepatocytes. This may also in part explain the lower number of cccDNA copies per cell frequently detected in patients [34,35,36] in comparison to woodchucks and ducks. Quantification of the exact number of cccDNA copies per cell, however, is very challenging because of possible variations from cell to cell or in different phases of the infection. Studies in woodchucks and ducks usually involve livers where infection is unrestricted and every hepatocyte is infected, while in patients only a proportion of hepatocytes appears to be infected. Nevertheless, in HBV-infected human liver chimeric mice with unrestricted infection of every hepatocyte, cccDNA counts per cell remain low (mostly just 1 to 3 copies/cell) even after long-term infection [37,38,39]. By employing humanized mice, we previously observed that intracellular cccDNA amplification mostly depended on new rounds of infection because treatment with the HBV entry inhibitor Myrcludex-B started in the spreading phase of infection efficiently blocked the increase of cccDNA per cell [40]. In this experimental setting, treatment of mice harboring low amounts of infected cells with Myrcludex-B not only blocked efficiently intrahepatic viral dissemination, but also appeared to hinder the increase of cccDNA contents in human hepatocytes that were already infected. Since increase of the cccDNA pool occurring via intracellular cccDNA amplification should have led to a detectable increase of total intrahepatic cccDNA amounts even in the absence of new infection events, we concluded that cccDNA amplification within already infected human hepatocytes barely took place in humanized mice in the absence of NTCP-mediated entry of new virions. Although this could be related to the model, the maintenance of a low cccDNA copy number per cell is in agreement with previous studies in human cells involving different systems [41]. In summary, these studies point out an unexpected contribution of the virus itself regarding its capacity to form the cccDNA and control its pool size.

## 4. HBV Treatment and Impact on the cccDNA

The half-life of the cccDNA has not been clearly defined. However, distinct in vitro studies indicated that the viral minichromosome is very stable in non-dividing human hepatocytes, where it appears to survive for the life span of the cell [31,42]. Thus, elimination of the cccDNA from the infected liver represents a major challenge and it seems to require either the destruction of the infected hepatocytes or the induction of substantial cccDNA destabilization. 

Currently approved treatments based on nucleoside analogs (NAs) effectively inhibit HBV reverse transcription leading to the reduction of viremia even below detection limits, but they do not target directly the cccDNA. Consequently, long-term antiviral therapy is needed to achieve significant reduction of the cccDNA pool [36,43,44,45,46,47,48,49]. In a large cohort of human immunodeficiency virus (HIV)-HBV co-infected patients under long-term tenofovir treatment, a continual decrease of cccDNA was observed progressing with a half-life of approximately 26 months in HBeAg-negative and nine months in HBeAg-positive patients [49]. The reasons for this cccDNA decrease are not entirely understood but will likely be caused by multiple factors such as the lack of incoming viruses from the blood and insufficient recycling of viral nucleocapsids to the nucleus due to the strong inhibition of viral DNA synthesis in the cytoplasm. Despite the absence of detectable viremia, cccDNA persistence within the hepatocytes is the reason for the relapse of viral activity after cessation of treatment with polymerase inhibitors in chronically infected individuals. Interestingly, recent studies showed that the occurrence of a transitory hepatic flare after stopping long-term nucleoside therapy was beneficial to treatment outcome in chronically HBV-infected patients [50,51]. It seems plausible that the abrupt stop of treatment in some individuals with long-term nucleoside/nucleotide analog (NA) therapy and the concomitant restoration of viral replication, might lead to the activation of the immune system resulting in the recognition and destruction of HBV-infected cells. According to this scenario, cell killing occurring during hepatic flares could significantly contribute to reduce cccDNA and viral antigen levels, both of which may be instrumental to gain immunological control.

Both cytopathic and non-cytopathic, cytokine-mediated mechanisms appear to contribute to cccDNA clearance [52,53,54,55] and cytokines involved in anti-HBV immunity were shown to inhibit HBV replication and even promote cccDNA destabilization [37,54,56,57,58,59,60]. Among these, interferon α (IFN-α), which is also used for the treatment of chronic HBV infection, was shown to accelerate pgRNA degradation and core particle decay in HBV transgenic mice [61,62,63,64]. Furthermore, experiments performed in vitro and in HBV-infected humanized mice revealed that IFNα can lower the levels of both pregenomic and subgenomic HBV RNAs by inducing epigenetic modifications of the histones bound to the cccDNA minichromosome [65]. In line with these observations, Chromatin Immunoprecipitation-Sequencing (ChIP-Seq) experiments demonstrated a reduction of active histone marks upon IFN-α administration, which could be recapitulated with a small molecule inhibitor of the responsible histone acetyltransferase indicating that reduced HBV replication and reduced levels of active histone marks are functionally linked [16]. These studies showed that by targeting cccDNA transcription, IFN-α can directly contribute to the decline of viral antigen amounts (HBeAg, HBsAg). Moreover, IFN-α administration was also shown to promote partial cccDNA degradation through the up-regulation of cytidine deaminases and nuclear factor κ-light-chain-enhancer of activated B cells (NFκB) pathways [54]. In patients, one year of combination therapy with polymerase inhibitor and IFN-α was shown to induce a stronger cccDNA reduction (2-log) than monotherapy with polymerase inhibitors alone [43,44]. However, despite such encouraging experimental evidences, interferon treatment induces immune clearance in only a minority of individuals [37,54,60,65]. As a consequence, HBV surface antigen (HBsAg) seroconversion rates remain low and the infection typically relapses after treatment cessation. Moreover, IFN-based therapy is often associated with systemic side-effects and contraindications, which represents a major drawback of this treatment.

## 5. The Contribution of Cell Division to HBV Resolution

HBV infection in immunocompetent adults generally results in a self-limited, transient liver disease, where viral control is achieved in more than 95% of adults. In general, resolution of infection typically requires effective viral recognition and concerted induction of innate and adaptive immune responses. Animal and clinical studies have demonstrated that in acute self-limited HBV infection, the CD8+ T cell and CD4+ T cell responses to HBV proteins are strong, polyclonal and multi-specific [66], whereas in chronic HBV infection immune responses appear weak and narrowly focused [67].

Immune CD8+ T cells and natural killer cells have the capacity not only to destroy the cccDNA together with the infected cell but also to induce proliferation of neighboring hepatocytes to compensate for the cell loss [67]. According to this scenario, studies in ducks that were treated with polymerase inhibitors indicated that cccDNA reduction was more profound in animals displaying higher hepatocyte proliferation rates [68]. Similarly, a significant cccDNA decrease was determined in cultured woodchuck hepatocytes infected with WHV when cell turnover was induced by cellular growth factors and viral replication was suppressed by NAs [42]. Based on data obtained in the growing livers of DHBV-infected ducklings treated with a nucleoside analogue a substantial proportion of cccDNA appeared to survive mitosis. However, because of considerable duck-to-duck variations and possibly incomplete inhibition of reverse transcription, a partial or even complete loss of cccDNA at mitosis could not be excluded in some of the animals [69]. Thus, studies with patient liver biopsies [70] and most animal hepadnavirus studies [55,68,69] pointed out an inverse relationship between hepatocyte turnover and cccDNA loads. Furthermore, the identification of uninfected cccDNA-negative cell clones containing “traces” of the infection in the form of viral integration demonstrated that cccDNA clearance without cell destruction can occur in chronically infected livers [71,72]. Thus, although the cccDNA minichromosome is very stable in non-dividing hepatocytes [31], killing of infected cells may be essential not only to eliminate infected cells but also to induce hepatocyte proliferation compensating for the loss of hepatocyte mass and leading to cccDNA destabilization in dividing hepatocytes. Both cytolytic and non-cytolytic cytokine-mediated mechanisms appear involved in cccDNA clearance during resolution of acute HBV infection, although their relative contributions, as well as the amount of hepatocyte destruction involved, are still debated. In particular, the fast recovery from acute self-limiting infection suggests that additional mechanisms may be involved to explain cccDNA clearance while the liver remains functional.

**Figure 1:**
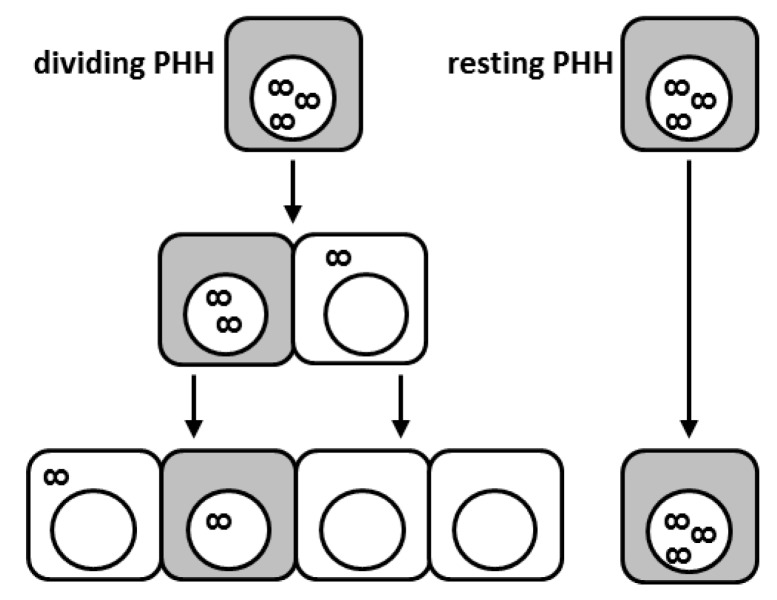
Proliferation of hepatitis B virus (HBV)-infected hepatocytes leads to covalently closed circular DNA (cccDNA) loss; however, HBV-infected cells display reduced proliferation capacities. Schematic illustration of the fate of individual cccDNA molecules during cell division. Shaded cells depict infected cells with active HBV replication. The infinity symbol depicts cccDNA. While proliferating hepatocytes (on the left-hand side) clear the infection through cccDNA dilution and eventually exclusion of cccDNA from the re-forming nucleus, some infected cells seem to be refractory to cell division (right-hand side). PHH: primary human hepatocyte.

Because the cccDNA is an extrachromosomal plasmid-like structure lacking centromeres, one could expect that upon hepatocyte division, the cccDNA molecules may become distributed in an unequal way among daughter cells or even get lost during mitosis (see Figure 1) [73,74]. In vitro studies indicated that episomal DNA molecules are mitotically instable and that cytosolic nucleases may be responsible for the rapid disposal of DNA molecules released into the cytoplasm [73]. In DHBV infection, the cccDNA appears to exist in a heterogeneous population of molecules with a full or a half complement of nucleosomes and therefore more condensed or open conformations [12]. It is tempting to speculate that distinct populations of cccDNA will differ in their susceptibility to degradation potentially also during cell division. We recently showed that in vivo proliferation of HBV-infected primary human hepatocytes leads to a strong cccDNA reduction in the liver of human chimeric mice [75]. Remarkably, cell division appeared to cause not only cccDNA dilution among daughter cells but also intrahepatic cccDNA loss. However, complete viral clearance was not achieved since HBV survived in sporadic non-proliferating human hepatocytes. As a consequence, virological markers rebounded as hepatocyte expansion relented in the experimental system used. Viral rebound was due to reinfection of quiescent primary human hepatocytes (PHHs) since treatment with the entry inhibitor Myrcludex-B blocked viral spread and intrahepatic cccDNA accumulation [75]. Notably, cccDNA reduction appeared even stronger in mice treated with the NA lamivudine, suggesting that inhibition of reverse transcription further accelerated cccDNA decrease also by impeding infection of the reforming nucleus by rcDNA-containing capsids still present in the cytoplasm. In any case, both treatments showed that replenishment of the cccDNA pool either via import of rcDNA from the cytoplasm after cell division or via de novo infection through circulating virions could not compensate for the great cccDNA loss provoked by cell division. Consequently, the cccDNA could be efficiently purged from the great majority of PHHs. Nevertheless, the persistence of very few, scattered non-proliferating cells expressing high levels of viral markers (HBcAg and HBV RNA) suggested that HBV infection may hinder cell division [76]. A lower proliferative capacity of hepatocytes was also determined in HBV-transgenic mice during liver regeneration possibly linking this perturbation of liver regeneration to HBV-related pathophysiology [76,77]. Nevertheless, even if apparently a paradox, it is indeed plausible that a decreased proliferative efficiency in the presence of high HBV replication levels may contribute to HBV-related oncogenesis, due to the different mechanisms by which viral components were shown to play a direct role in carcinogenesis [78]. It remains to be determined whether HBV actively inhibits cell division to escape dilution of the cccDNA pool among progeny cells or whether HBV survives in a certain hepatocyte subpopulation, which might be refractory to cell division. Yet, a reduced ability of HBV-positive cells to divide might have important clinical implications, since cells refractory to cell division may serve as reservoir of infection (see Figure 1). Thus, the development of therapeutic strategies efficiently targeting such persisting HBV-producing cells may be fundamental to achieve viral elimination [79]. The strong cccDNA drop caused by cell division suggests that curative therapeutic approaches may necessitate the involvement of controlled destruction of infected cells (i.e., by boosting immune responses) also to promote cccDNA loss in surviving proliferating hepatocytes. At the same time, strategies preventing HBV entry and/or suppressing viral replication would prevent re-infection of cured hepatocytes.

Such findings point out the important role that immune modulating factors play in reducing cccDNA loads and activity. We speculate that both direct killing of infected cells and compensatory proliferation of HBV-infected hepatocytes play a key synergistic role in the process of resolving acute hepatitis. This process might have also been functional in chronically HBV infected patients, which were reported to achieve more beneficial treatment outcomes when the stopping of long-term NA therapy was accompanied with a transitory hepatic flare [50,51,80].

## 6. Conclusions

In sum, even if elimination of the cccDNA from the infected liver remains challenging and more research is needed to develop therapeutic approaches boosting HBV-specific immune responses or agents directly targeting the cccDNA, cell division appears to be a natural Achilles’ heel in HBV persistence. At the same time, key factors produced by immune cells, like IFNs and tumor necrosis factor α, can further promote non-cytolytic inhibition of HBV replication and contribute to cccDNA destabilization [56]. The fact that even after a resolved acute infection low amounts of cccDNA molecules are detected in the liver of patients [81], raises hope that not the entire intrahepatic cccDNA reservoir needs to be eliminated to gain immunological control and resolve the infection. We hypothesize that if a sufficient amount of HBV-infected cells will be eliminated and even more cccDNA will be degraded through compensatory proliferation and non-cytolytic immune factors, the immune system should be able to resolve chronic HBV infection.

## References

[1] Zeisel M.B., Lucifora J., Mason W.S., Sureau C., Beck J., Levrero M., Kann M., Knolle P.A., Benkirane M., Durantel D. (2015). Towards an HBV cure: State-of-the-art and unresolved questions—Report of the anrs workshop on HBV cure. Gut.

[2] Yan H., Zhong G., Xu G., He W., Jing Z., Gao Z., Huang Y., Qi Y., Peng B., Wang H. (2012). Sodium taurocholate cotransporting polypeptide is a functional receptor for human hepatitis B and D virus. eLife.

[3] Rabe B., Glebe D., Kann M. (2006). Lipid-mediated introduction of hepatitis B virus capsids into nonsusceptible cells allows highly efficient replication and facilitates the study of early infection events. J. Virol..

[4] Kann M., Schmitz A., Rabe B. (2007). Intracellular transport of hepatitis B virus. World J. Gastroenterol..

[5] Guo H., Xu C., Zhou T., Block T.M., Guo J.T. (2012). Characterization of the host factors required for hepadnavirus covalently closed circular (ccc) DNA formation. PLoS ONE.

[6] Schreiner S., Nassal M. (2017). A role for the host DNA damage response in hepatitis B virus cccDNA formation-and beyond?. Viruses.

[7] Guo H., Jiang D., Zhou T., Cuconati A., Block T.M., Guo J.T. (2007). Characterization of the intracellular deproteinized relaxed circular DNA of hepatitis B virus: An intermediate of covalently closed circular DNA formation. J. Virol..

[8] Nassal M. (2015). HBV cccDNA: Viral persistence reservoir and key obstacle for a cure of chronic hepatitis B. Gut.

[9] Qi Y., Gao Z., Xu G., Peng B., Liu C., Yan H., Yao Q., Sun G., Liu Y., Tang D. (2016). DNA polymerase kappa is a key cellular factor for the formation of covalently closed circular DNA of hepatitis B virus. PLoS Pathog..

[10] Koniger C., Wingert I., Marsmann M., Rosler C., Beck J., Nassal M. (2014). Involvement of the host DNA—Repair enzyme TDP2 in formation of the covalently closed circular DNA persistence reservoir of hepatitis B viruses. Proc. Natl. Acad. Sci. USA.

[11] Cui X., McAllister R., Boregowda R., Sohn J.A., Cortes Ledesma F., Caldecott K.W., Seeger C., Hu J. (2015). Does tyrosyl DNA phosphodiesterase-2 play a role in hepatitis B virus genome repair?. PLoS ONE.

[12] Newbold J.E., Xin H., Tencza M., Sherman G., Dean J., Bowden S., Locarnini S. (1995). The covalently closed duplex form of the hepadnavirus genome exists in situ as a heterogeneous population of viral minichromosomes. J. Virol..

[13] Levrero M., Pollicino T., Petersen J., Belloni L., Raimondo G., Dandri M. (2009). Control of cccDNA function in hepatitis B virus infection. J. Hepatol..

[14] Quasdorff M., Protzer U. (2010). Control of hepatitis B virus at the level of transcription. J. Viral. Hepat..

[15] Pollicino T., Belloni L., Raffa G., Pediconi N., Squadrito G., Raimondo G., Levrero M. (2006). Hepatitis B virus replication is regulated by the acetylation status of hepatitis B virus cccDNA-bound H3 and H4 histones. Gastroenterology.

[16] Tropberger P., Mercier A., Robinson M., Zhong W., Ganem D.E., Holdorf M. (2015). Mapping of histone modifications in episomal HBV cccDNA uncovers an unusual chromatin organization amenable to epigenetic manipulation. Proc. Natl. Acad. Sci. USA.

[17] Bock C.T., Schwinn S., Locarnini S., Fyfe J., Manns M.P., Trautwein C., Zentgraf H. (2001). Structural organization of the hepatitis B virus minichromosome. J. Mol. Biol..

[18] Belloni L., Pollicino T., De Nicola F., Guerrieri F., Raffa G., Fanciulli M., Raimondo G., Levrero M. (2009). Nuclear HBx binds the HBV minichromosome and modifies the epigenetic regulation of cccDNA function. Proc. Natl. Acad. Sci. USA.

[19] Lucifora J., Arzberger S., Durantel D., Belloni L., Strubin M., Levrero M., Zoulim F., Hantz O., Protzer U. (2011). Hepatitis B virus x protein is essential to initiate and maintain virus replication after infection. J. Hepatol..

[20] Decorsiere A., Mueller H., van Breugel P.C., Abdul F., Gerossier L., Beran R.K., Livingston C.M., Niu C., Fletcher S.P., Hantz O. (2016). Hepatitis B virus X protein identifies the Smc5/6 complex as a host restriction factor. Nature.

[21] Murphy C.M., Xu Y., Li F., Nio K., Reszka-Blanco N., Li X., Wu Y., Yu Y., Xiong Y., Su L. (2016). Hepatitis B virus X protein promotes degradation of Smc5/6 to enhance HBV replication. Cell Rep..

[22] Wieland S., Thimme R., Purcell R.H., Chisari F.V. (2004). Genomic analysis of the host response to hepatitis B virus infection. Proc. Natl. Acad. Sci. USA.

[23] Dunn C., Peppa D., Khanna P., Nebbia G., Jones M., Brendish N., Lascar R.M., Brown D., Gilson R.J., Tedder R.J. (2009). Temporal analysis of early immune responses in patients with acute hepatitis B virus infection. Gastroenterology.

[24] Lebosse F., Testoni B., Fresquet J., Facchetti F., Galmozzi E., Fournier M., Hervieu V., Berthillon P., Berby F., Bordes I. (2017). Intrahepatic innate immune response pathways are downregulated in untreated chronic hepatitis B. J. Hepatol..

[25] Nassal M. (2008). Hepatitis B viruses: Reverse transcription a different way. Virus Res..

[26] Hu J., Liu K. (2017). Complete and incomplete hepatitis B virus particles: Formation, function, and application. Viruses.

[27] Wang J., Shen T., Huang X., Kumar G.R., Chen X., Zeng Z., Zhang R., Chen R., Li T., Zhang T. (2016). Serum hepatitis B virus RNA is encapsidated pregenome RNA that may be associated with persistence of viral infection and rebound. J. Hepatol..

[28] Giersch K., Allweiss L., Volz T., Dandri M., Lutgehetmann M. (2017). Serum HBV pgRNA as a clinical marker for cccDNA activity. J. Hepatol..

[29] Van Bommel F., Bartens A., Mysickova A., Hofmann J., Kruger D.H., Berg T., Edelmann A. (2015). Serum hepatitis B virus RNA levels as an early predictor of hepatitis B envelope antigen seroconversion during treatment with polymerase inhibitors. Hepatology.

[30] Zhang Y.Y., Zhang B.H., Theele D., Litwin S., Toll E., Summers J. (2003). Single-cell analysis of covalently closed circular DNA copy numbers in a hepadnavirus-infected liver. Proc. Natl. Acad. Sci. USA.

[31] Moraleda G., Saputelli J., Aldrich C.E., Averett D., Condreay L., Mason W.S. (1997). Lack of effect of antiviral therapy in nondividing hepatocyte cultures on the closed circular DNA of woodchuck hepatitis virus. J. Virol..

[32] Hantz O., Parent R., Durantel D., Gripon P., Guguen-Guillouzo C., Zoulim F. (2009). Persistence of the hepatitis B virus covalently closed circular DNA in heparg human hepatocyte-like cells. J. Gen. Virol..

[33] Kock J., Rosler C., Zhang J.J., Blum H.E., Nassal M., Thoma C. (2010). Generation of covalently closed circular DNA of hepatitis B viruses via intracellular recycling is regulated in a virus specific manner. PLoS Pathog..

[34] Laras A., Koskinas J., Dimou E., Kostamena A., Hadziyannis S.J. (2006). Intrahepatic levels and replicative activity of covalently closed circular hepatitis B virus DNA in chronically infected patients. Hepatology.

[35] Volz T., Lutgehetmann M., Wachtler P., Jacob A., Quaas A., Murray J.M., Dandri M., Petersen J. (2007). Impaired intrahepatic hepatitis B virus productivity contributes to low viremia in most HBeAg-negative patients. Gastroenterology.

[36] Werle-Lapostolle B., Bowden S., Locarnini S., Wursthorn K., Petersen J., Lau G., Trepo C., Marcellin P., Goodman Z., Delaney W.E. (2004). Persistence of cccDNA during the natural history of chronic hepatitis B and decline during adefovir dipivoxil therapy. Gastroenterology.

[37] Allweiss L., Volz T., Lutgehetmann M., Giersch K., Bornscheuer T., Lohse A.W., Petersen J., Ma H., Klumpp K., Fletcher S.P. (2014). Immune cell responses are not required to induce substantial hepatitis B virus antigen decline during pegylated interferon-α administration. J. Hepatol..

[38] Lutgehetmann M., Bornscheuer T., Volz T., Allweiss L., Bockmann J.H., Pollok J.M., Lohse A.W., Petersen J., Dandri M. (2011). Hepatitis B virus limits response of human hepatocytes to interferon-α in chimeric mice. Gastroenterology.

[39] Lutgehetmann M., Mancke L.V., Volz T., Helbig M., Allweiss L., Bornscheuer T., Pollok J.M., Lohse A.W., Petersen J., Urban S. (2012). Humanized chimeric uPAa mouse model for the study of hepatitis B and D virus interactions and preclinical drug evaluation. Hepatology.

[40] Volz T., Allweiss L., Ben M.M., Warlich M., Lohse A.W., Pollok J.M., Alexandrov A., Urban S., Petersen J., Lutgehetmann M. (2013). The entry inhibitor myrcludex-B efficiently blocks intrahepatic virus spreading in humanized mice previously infected with hepatitis B virus. J. Hepatol..

[41] Lucifora J., Protzer U. (2016). Attacking hepatitis B virus cccDNA—The holy grail to hepatitis B cure. J. Hepatol..

[42] Dandri M., Burda M.R., Will H., Petersen J. (2000). Increased hepatocyte turnover and inhibition of woodchuck hepatitis B virus replication by adefovir in vitro do not lead to reduction of the closed circular DNA. Hepatology.

[43] Wursthorn K., Buggisch P., Lutgehetmann M., Zollner B., Petersen J. (2006). Temporary HBV resolution in an HIV-coinfected patient during HBV-directed combination therapy followed by relapse of HBV. Antivir. Ther..

[44] Lutgehetmann M., Volzt T., Quaas A., Zankel M., Fischer C., Dandri M., Petersen J. (2008). Sequential combination therapy leads to biochemical and histological improvement despite low ongoing intrahepatic hepatitis B virus replication. Antivir. Ther..

[45] Bowden S., Locarnini S., Chang T.T., Chao Y.C., Han K.H., Gish R.G., de Man R.A., Yu M., Llamoso C., Tang H. (2015). Covalently closed-circular hepatitis B virus DNA reduction with entecavir or lamivudine. World J. Gastroenterol..

[46] Sung J.J., Wong M.L., Bowden S., Liew C.T., Hui A.Y., Wong V.W., Leung N.W., Locarnini S., Chan H.L. (2005). Intrahepatic hepatitis B virus covalently closed circular DNA can be a predictor of sustained response to therapy. Gastroenterology.

[47] Wong D.K., Yuen M.F., Ngai V.W., Fung J., Lai C.L. (2006). One-year entecavir or lamivudine therapy results in reduction of hepatitis B virus intrahepatic covalently closed circular DNA levels. Antivir. Ther..

[48] Zheng Q., Zhu Y.Y., Chen J., Liu Y.R., You J., Dong J., Zeng D.W., Gao L.Y., Chen L.H., Jiang J.J. (2014). Decline in intrahepatic cccDNA and increase in immune cell reactivity after 12 weeks of antiviral treatment were associated with HBeAg loss. J. Viral. Hepat..

[49] Boyd A., Lacombe K., Lavocat F., Maylin S., Miailhes P., Lascoux-Combe C., Delaugerre C., Girard P.M., Zoulim F. (2016). Decay of ccc-DNA marks persistence of intrahepatic viral DNA synthesis under tenofovir in HIV-HBV co-infected patients. J. Hepatol..

[50] Hadziyannis S.J., Sevastianos V., Rapti I., Vassilopoulos D., Hadziyannis E. (2012). Sustained responses and loss of hbsag in HBeAg-negative patients with chronic hepatitis B who stop long-term treatment with adefovir. Gastroenterology.

[51] Chang M.L., Liaw Y.F., Hadziyannis S.J. (2015). Systematic review: Cessation of long-term nucleos(t)ide analogue therapy in patients with hepatitis B e antigen-negative chronic hepatitis B. Aliment. Pharmacol. Ther..

[52] Chisari F.V. (1997). Cytotoxic T cells and viral hepatitis. J. Clin. Investig..

[53] Guidotti L.G., Rochford R., Chung J., Shapiro M., Purcell R., Chisari F.V. (1999). Viral clearance without destruction of infected cells during acute HBV infection. Science.

[54] Lucifora J., Xia Y., Reisinger F., Zhang K., Stadler D., Cheng X., Sprinzl M.F., Koppensteiner H., Makowska Z., Volz T. (2014). Specific and nonhepatotoxic degradation of nuclear hepatitis B virus cccDNA. Science.

[55] Mason W.S., Litwin S., Xu C., Jilbert A.R. (2007). Hepatocyte turnover in transient and chronic hepadnavirus infections. J. Viral. Hepat..

[56] Xia Y., Stadler D., Lucifora J., Reisinger F., Webb D., Hosel M., Michler T., Wisskirchen K., Cheng X., Zhang K. (2016). Interferon-γ and tumor necrosis factor-α produced by T cells reduce the HBV persistence form, cccDNA, without cytolysis. Gastroenterology.

[57] Thimme R., Wieland S., Steiger C., Ghrayeb J., Reimann K.A., Purcell R.H., Chisari F.V. (2003). CD8(+) T cells mediate viral clearance and disease pathogenesis during acute hepatitis B virus infection. J. Virol..

[58] Hosel M., Quasdorff M., Wiegmann K., Webb D., Zedler U., Broxtermann M., Tedjokusumo R., Esser K., Arzberger S., Kirschning C.J. (2009). Not interferon, but interleukin-6 controls early gene expression in hepatitis B virus infection. Hepatology.

[59] Palumbo G.A., Scisciani C., Pediconi N., Lupacchini L., Alfalate D., Guerrieri F., Calvo L., Salerno D., Di Cocco S., Levrero M. (2015). IL6 inhibits HBV transcription by targeting the epigenetic control of the nuclear cccDNA minichromosome. PLoS ONE.

[60] Liang T.J., Block T.M., McMahon B.J., Ghany M.G., Urban S., Guo J.T., Locarnini S., Zoulim F., Chang K.M., Lok A.S. (2015). Present and future therapies of hepatitis B: From discovery to cure. Hepatology.

[61] Wieland S.F., Guidotti L.G., Chisari F.V. (2000). Intrahepatic induction of α/β interferon eliminates viral RNA-containing capsids in hepatitis B virus transgenic mice. J. Virol..

[62] Wieland S.F., Eustaquio A., Whitten-Bauer C., Boyd B., Chisari F.V. (2005). Interferon prevents formation of replication-competent hepatitis B virus RNA-containing nucleocapsids. Proc. Natl. Acad. Sci. USA.

[63] Uprichard S.L., Wieland S.F., Althage A., Chisari F.V. (2003). Transcriptional and posttranscriptional control of hepatitis B virus gene expression. Proc. Natl. Acad. Sci. USA.

[64] Xu C., Guo H., Pan X.B., Mao R., Yu W., Xu X., Wei L., Chang J., Block T.M., Guo J.T. (2010). Interferons accelerate decay of replication-competent nucleocapsids of hepatitis B virus. J. Virol..

[65] Belloni L., Allweiss L., Guerrieri F., Pediconi N., Volz T., Pollicino T., Petersen J., Raimondo G., Dandri M., Levrero M. (2012). IFN-α inhibits HBV transcription and replication in cell culture and in humanized mice by targeting the epigenetic regulation of the nuclear cccDNA minichromosome. J. Clin. Investig..

[66] Guidotti L.G., Chisari F.V. (2001). Noncytolytic control of viral infections by the innate and adaptive immune response. Annu. Rev. Immunol..

[67] Bertoletti A., Ferrari C. (2012). Innate and adaptive immune responses in chronic hepatitis B virus infections: Towards restoration of immune control of viral infection. Gut.

[68] Addison W.R., Walters K.A., Wong W.W., Wilson J.S., Madej D., Jewell L.D., Tyrrell D.L. (2002). Half-life of the duck hepatitis B virus covalently closed circular DNA pool in vivo following inhibition of viral replication. J. Virol..

[69] Reaiche-Miller G.Y., Thorpe M., Low H.C., Qiao Q., Scougall C.A., Mason W.S., Litwin S., Jilbert A.R. (2013). Duck hepatitis B virus covalently closed circular DNA appears to survive hepatocyte mitosis in the growing liver. Virology.

[70] Mason W.S., Liu C., Aldrich C.E., Litwin S., Yeh M.M. (2010). Clonal expansion of normal-appearing human hepatocytes during chronic hepatitis B virus infection. J. Virol..

[71] Mason W.S., Jilbert A.R., Summers J. (2005). Clonal expansion of hepatocytes during chronic woodchuck hepatitis virus infection. Proc. Natl. Acad. Sci. USA.

[72] Mason W.S., Low H.C., Xu C., Aldrich C.E., Scougall C.A., Grosse A., Clouston A., Chavez D., Litwin S., Peri S. (2009). Detection of clonally expanded hepatocytes in chimpanzees with chronic hepatitis B virus infection. J. Virol..

[73] Lechardeur D., Sohn K.J., Haardt M., Joshi P.B., Monck M., Graham R.W., Beatty B., Squire J., O’Brodovich H., Lukacs G.L. (1999). Metabolic instability of plasmid DNA in the cytosol: A potential barrier to gene transfer. Gene Ther..

[74] Wang X., Le N., Denoth-Lippuner A., Barral Y., Kroschewski R. (2016). Asymmetric partitioning of transfected DNA during mammalian cell division. Proc. Natl. Acad. Sci. USA.

[75] Allweiss L., Volz T., Giersch K., Kah J., Raffa G., Petersen J., Lohse A.W., Beninati C., Pollicino T., Urban S. (2017). Proliferation of primary human hepatocytes and prevention of hepatitis B virus reinfection efficiently deplete nuclear cccDNA in vivo. Gut.

[76] Wu B.K., Li C.C., Chen H.J., Chang J.L., Jeng K.S., Chou C.K., Hsu M.T., Tsai T.F. (2006). Blocking of g1/s transition and cell death in the regenerating liver of hepatitis B virus X protein transgenic mice. Biochem. Biophys. Res. Commun..

[77] Quetier I., Brezillon N., Duriez M., Massinet H., Giang E., Ahodantin J., Lamant C., Brunelle M.N., Soussan P., Kremsdorf D. (2013). Hepatitis B virus HBx protein impairs liver regeneration through enhanced expression of IL-6 in transgenic mice. J. Hepatol..

[78] Ringelhan M., O’Connor T., Protzer U., Heikenwalder M. (2015). The direct and indirect roles of HBV in liver cancer: Prospective markers for hcc screening and potential therapeutic targets. J. Pathol..

[79] Koh S., Bertoletti A. (2015). Circumventing failed antiviral immunity in chronic hepatitis B virus infection: Triggering virus-specific or innate-like t cell response?. Med. Microbiol. Immunol..

[80] Honer Zu Siederdissen C., Rinker F., Maasoumy B., Wiegand S.B., Filmann N., Falk C.S., Deterding K., Port K., Mix C., Manns M.P. (2016). Viral and host responses after stopping long-term nucleos(t)ide analogue therapy in HBeAg-negative chronic hepatitis B. J. Infect. Dis..

[81] Rehermann B., Ferrari C., Pasquinelli C., Chisari F.V. (1996). The hepatitis B virus persists for decades after patients’ recovery from acute viral hepatitis despite active maintenance of a cytotoxic T-lymphocyte response. Nat. Med..

